# «I Do Not Have Time»—Is This the End of Peer Review in Public Health Sciences?

**DOI:** 10.3389/phrs.2022.1605407

**Published:** 2022-11-17

**Authors:** Nino Künzli, Anke Berger, Katarzyna Czabanowska, Raquel Lucas, Andrea Madarasova Geckova, Sarah Mantwill, Olaf von dem Knesebeck

**Affiliations:** ^1^ Swiss Tropical and Public Health Institute, Allschwil, Switzerland; ^2^ Swiss School of Public Health, Zürich, Switzerland; ^3^ University of Basel, Basel, Switzerland; ^4^ Department of International Health, Care and Public Health Research Institute (CAPHRI), Maastricht University, Maastricht, Netherlands; ^5^ Epidemiology Research Unit, Institute of Public Health, University of Porto, Porto, Portugal; ^6^ Department of Health Psychology and Research Methodology, Pavol Jozef Šafárik University in Košice, Košice, Slovakia; ^7^ Department of Health Sciences and Medicine, University of Lucerne, Lucerne, Switzerland; ^8^ Institute of Medical Sociology, University of Hamburg, Hamburg, Germany

**Keywords:** peer review crisis, reviewer declines, pre-publication peer review, post-publication peer review, reviewer incentives, public health science journals

At the end of this Editorial, we ask you to take a few minutes to respond to our short anonymous online query. If you have no time to read this Editorial about the possible collapse of the peer review system, please just respond to the survey. But we—the editors in chief and managing editor of the International Journal of Public Health (IJPH) and the Public Health Reviews (PHR)—hope you dedicate the time to read this Editorial about the peer review crisis in public health sciences.

In a 2019 workshop of the Swiss School of Public Health (SSPH+)—the owner of the IJPH and PHR—all participants agreed that our journals should continue with thorough pre-publication peer review, including revisions, point-by-point responses and final decisions made by science editors, to strengthen the quality of publications. But soon after, the explosion in COVID-19 research caused an unprecedented increase in submissions and thus demand for peer review in health science journals [1–3]. During the first 6 months of the pandemic, total publications and COVID-19 related publications increased exponentially [2]. Submissions to JAMA almost tripled [4] and to IJPH more than tripled. Elsevier’s health and medical journals saw a 63% increase in submissions between February and May 2020, compared to the same period in 2019 [3]. The publication rates of all peer reviewed public-health related articles increased by 25% from 2019 to 2020, and by 21% from 2020 to 2021, exceeding the annual publication growth rates before the pandemic which were 4, 15, 9 and 19% from 2016 until 2019 (Web of Science, category Public, Environmental and Occupational Health).

The growth in publications has brought the peer review system to the edge, as the commitment of scientists did not keep pace with the increase in manuscripts. In Elsevier’s health and medical journals the increase in peer review invitation acceptances from 2019 to 2020 (February to May periods) was about 50% lower than the increase in submissions [3]. The records of IJPH provide sobering facts, too. “I do not have time” has become a leading response—and even worse: the majority does not respond at all. In 2021, 53% of (repeated) invitations for peer review remained un-answered, whereas 38% actively declined. Both, in 2021 and 2022, only 9% of all invited reviewers agreed to provide a review. In contrast, during the years prior to the pandemic, we observed a rather stable rate of 35%–40% who would agree and deliver a review report.

The consequences of this crisis are equally bad for our authors and editors. First, the endless search for reviewers has slowed down the publishing process substantially. Second, editors have to resort to the release of automatic “mass-invitations” to 20+ potential reviewers at a time and to remind those repeatedly, thus, flooding the stressed community with even more invitation emails. Third, automated search strategies require artificial big-data search engines. As those are of limited intelligence yet, a rather high rate of 25%—probably the tip of an iceberg—of active decliners tell us “this is not my field.” Fourth, we are forced to discuss whether and under what conditions final decisions should be based on the feedbacks of only one review. And last but not least, the workload for our handling editors has also increased substantially, which in turn triggers their resistance to handle manuscripts.

The simple truth is: if we collectively “do not have time” to review manuscripts, there will be no peer review anymore. Thus, key questions emerge. Does public health science serve authorities and the public sufficiently if research is published without formal pre-publication peer review? Is post-publication peer review also appropriate to promote good public health sciences? Will publishing on pre-print servers become the new standard given the abundance of innovation in this field [5]. In the absence of clear evidence for the opposite, we strongly believe that the multi-disciplinary public health sciences profit from pre-publication peer review. Thus, we need a solution to address the crisis.

Given the ubiquity and magnitude of the problem, we call for concerted strategies also of publishers to resolve it together with journal editors as they cannot do it alone. The literature discusses various incentives for reviewers, among them discounts on publishers’ products, certificates, recognizing the best reviewers, increasing diversity in the peer-review process and financial incentives [5–8]. The development of reviewer recognition platforms (ORCID, Clarivate’s reviewer recognition platform) is a positive example.

From a quantitative perspective, the solution looks rather straightforward in our typically multi-authored science: if researchers agree to review as many papers per year as they write as first or last author, the crisis would resolve. Indeed, the trends in scientific publishing where the number of publications doubles every 10 years whilst the number of scientists increases by only 21% [7] call for such level of commitment. When reviewers are asked, a vast majority (85%) find recognition and training will improve the efficacy of peer review and, that universities and research funders should explicitly require and recognize the reviewing work which should be career enhancing [9].

Our two journals have adopted a feature to remove at least barriers of the peer review mode (blinded or open) on reviewers’ willingness to review: while we run peer reviews double blind to minimize biases, we leave it up to the reviewers to decide after peer review if they would like to publish their name, the review report or both with the published article. We are aware that reviews of rejected articles are not recognized this way and therefore, encourage reviewers to register their reviews on reviewer recognition platforms. We invite excellent reviewers who provided relevant information beyond the content of an article to comment in an Editorial. We publish reviewer acknowledgments. And on request we issue reviewer certificates. We offer an online course to train junior researchers in editorial tasks and peer reviewing. However, this is not enough.

As a non-profit society journal, we wonder whether our research community is more open to review for society journals as compared to the many for-profit journals owned by publishers and their shareholders. Or does it matter who the publisher—rather than the owner—of the journal is? Alternatively, is the Impact Factor of a journal or its open access status relevant for scientists’ decision to review or decline? IJPH has a Q1 Impact Factor of 5.1 and PHR’s Q1 CiteScore of 9.6 is promising for its first Impact Factor to be obtained next year. Both journals publish gold open access. Do scientists consider whether journals promote early career researchers and researchers from low and middle- income countries like we do with our Young Researcher Editorial Series (YRE) in IJPH and the Globequity APC waiver program?

In an attempt to better understand the requirements of reviewers of PHR and IJPH we reach out to you. We would greatly appreciate your answers to a few questions that will guide our decisions to foster your willingness to review for IJPH and PHR.

Please kindly follow this link https://ssphplus.ch/en/ssph-journals/survey-peer-review/ to our query.

Do you have time to counter the peer review crisis? We very much hope!
